# Context-aware genomic surveillance reveals hidden transmission of a carbapenemase-producing *Klebsiella pneumoniae*

**DOI:** 10.1099/mgen.0.000741

**Published:** 2021-12-16

**Authors:** Adrian Viehweger, Christian Blumenscheit, Norman Lippmann, Kelly L. Wyres, Christian Brandt, Jörg B. Hans, Martin Hölzer, Luiz Irber, Sören Gatermann, Christoph Lübbert, Mathias W. Pletz, Kathryn E. Holt, Brigitte König

**Affiliations:** ^1^​ Institute of Medical Microbiology and Virology, University Hospital Leipzig, Leipzig, Germany; ^2^​ ZBS 6: Proteomics and Spectroscopy, Robert Koch Institute, Berlin, Germany; ^3^​ Department of Infectious Diseases, Central Clinical School, Monash University, Melbourne, Australia; ^4^​ Institute for Infectious Diseases and Infection Control, Jena University Hospital, Jena, Germany; ^5^​ National Reference Center for multidrug-resistant Gram-negative bacteria, Department for Medical Microbiology, Ruhr-University Bochum, Bochum, Germany; ^6^​ Methodology and Research Infrastructure, MF1 Bioinformatics, Robert Koch Institute, Berlin, Germany; ^7^​ Department of Population Health and Reproduction, University of California, Davis, Davis, California, USA; ^8^​ Division of Infectious Diseases and Tropical Medicine, Department of Medicine II, University Hospital Leipzig, Leipzig, Germany; ^9^​ Department of Infection Biology, London School of Hygiene and Tropical Medicine, London, UK

**Keywords:** genomic surveillance, meta-analysis, antimicrobial resistance, KPC, plasmids, colistin

## Abstract

Genomic surveillance can inform effective public health responses to pathogen outbreaks. However, integration of non-local data is rarely done. We investigate two large hospital outbreaks of a carbapenemase-carrying *Klebsiella pneumoniae* strain in Germany and show the value of contextual data. By screening about 10 000 genomes, over 400 000 metagenomes and two culture collections using *in silico* and *in vitro* methods, we identify a total of 415 closely related genomes reported in 28 studies. We identify the relationship between the two outbreaks through time-dated phylogeny, including their respective origin. One of the outbreaks presents extensive hidden transmission, with descendant isolates only identified in other studies. We then leverage the genome collection from this meta-analysis to identify genes under positive selection. We thereby identify an inner membrane transporter (*ynjC*) with a putative role in colistin resistance. Contextual data from other sources can thus enhance local genomic surveillance at multiple levels and should be integrated by default when available.

## Data Summary

All data and metadata used in the analyses have been deposited with the *Open Science Foundation* (OSF) under project ID n78q3. Extensive metadata on all samples used in the analyses is available there and in the Supplementary Material (Table S1, available in the online version of this article), including curated phenotype data on colistin resistance (Table S2). In addition, all genomes collected specifically for this study have been deposited with *NCBI GenBank* under project ID PRJNA742413. For all contextual genome data, please refer to the corresponding studies (Table S1). Table S3 presents all genomes excluded manually from the analysis (see Methods). Table S4 holds isolate feature annotations from kleborate such as sequence type and a selection of resistance determinants. Table S5 provides metadata for all genomes that did not pass the tiered quality protocol (see Methods) and were thus not included in the analyses.

**Impact Statement**

Usually, hospital outbreaks are analysed in isolation. However, here we demonstrate how to screen publicly available genomes, metagenomes and culture isolates. We place a local multidrug-resistant *bla*_KPC-2_ -carrying strain of *Klebsiella pneumoniae* in a larger genomic context. This context provides important details about the origin and distribution of the outbreak-causing strain and reveals hidden transmission far from where the outbreak occurred. Contextual genomes can also facilitate inquiries into genomic determinants of resistance. We demonstrate this by identifying a new putative target for colistin resistance, an antibiotic of last resort, thereby using the collected data to move beyond the local outbreak in a step-function improvement.

## Introduction

Multiresistant strains of *Klebsiella pneumoniae* (Kp) are a global health threat [1]. Among all known resistance mechanisms, carbapenemases are one of the most concerning, as carbapenem antibiotics are considered a last-line drug. These enzymes are typically encoded on mobile genetic elements such as the Tn*4401* transposon [2], which mediates transfer between plasmids [3] and bacterial species [4]. Furthermore, the prevalence of carbapenemase-producing Kp has increased in recent years [5], leaving only few antibiotics such as colistin to treat those cases. Such pathogen spread can be prevented by molecular surveillance and derived public health measures: isolate genomes reveal transmission routes by accumulating characteristic mutations, from which ancestry can be inferred through time-dated phylogeny [6].

While it has become standard practice to reconstruct such phylogenies of within-hospital outbreaks [5, 7], few studies assess ‘contextual’ information, i.e. genome sequences from isolates that were not part of the local outbreak but closely related. From a public health perspective, this is suboptimal. While many larger hospitals run screening programmes to detect the carriage of resistant strains on admission [8, 9], peripheral institutions rarely do. However, there is a significant transfer of patients, e.g. from operation theatre to rehabilitation centre or from one country to another. For an outbreak investigation with only local scope, these boundary-crossing transmission events remain hidden.

Here we reanalyze a large outbreak at the University Hospital Leipzig (UHL) from 2010 to 2013 [10] in light of new data from a nearby institution, which experienced an outbreak with a closely related, albeit non-descending, strain. We performed a genomic meta-analysis to link both outbreaks, discovering hundreds of related genomes distributed across 28 different studies. We identify the likely sources of both outbreaks and illustrate hidden transmission across study boundaries. Only the integration of data from several sources provided a ‘complete picture’. However, we highlight several obstacles that need to be addressed before cross-boundary genomic surveillance can work in practice.

Beyond epidemiology, we show how outbreak meta-analyses can generate new hypotheses about host adaptation and antimicrobial resistance: the genomes under study underly similar selective pressures, such as treatment with colistin, an antibiotic of last resort. Thus, recurring mutations in the same gene(s) but across different genomes can signal putative causes for an observed phenotype, such as colistin resistance [11]. For colistin, several such inducible genomic changes have been described that mediate resistance [12]. Nevertheless, the exact mechanisms remain incompletely understood and seem to be multifactorial [13]. We show how contextual data can be leveraged to generate hypotheses about putative factors contributing to colistin resistance.

## Methods

### Culture and sequencing

All samples were streaked on CHROMagar KPC chromogenic agar plates (CHROMagar, Paris, France). *KPC* carriage of isolates was confirmed using PCR. In total, 142 Kp-1 isolates had been collected from 105 patients in a previous investigation [10] and were complemented in the present study with an additional ten isolates discovered using PCR screening of two culture collections (see below) and 13 samples collected from Kp-2. All of the isolates were sequenced using short reads (Illumina). Short-read sequencing for isolates from other studies is described in the respective publications (Table S1). For genomes sequenced for the current study, a read length of 150 bases (paired-end) was used on an Illumina MiSeq sequencer. The libraries were constructed using a previously established protocol [14]. For the current study, 28 Kp-1 samples were additionally sequenced using long reads (Nanopore) to enable hybrid assembly (see below). DNA extraction for Nanopore sequencing and quality control was done as reported elsewhere [15]. Care must be taken, especially for Nanopore sequencing, not to damage the extracted DNA to achieve a sizeable median fragment length (target 8 kb) for sequencing to be effective. Nanopore sequencing was performed using the MinION sequencer and the 1D ligation library kit (LSK109) on an R9.4 flow cell (all Oxford Nanopore Technologies, ONT).

### *In silico* screening of isolate and metagenomes

Through screening, our aim was to collect as many genomes as possible with a putative relation to the outbreak clone Kp-1, yielding a total of 9409 genomes. From NCBI *RefSeq*, we retrieved all 9163 genomes that were labelled as *Klebsiella pneumoniae* (Taxonomy ID: 573, last accessed 1 August 2020) [16]. In a comprehensive literature search using the search terms ‘KPC, *Klebsiella pneumoniae *, outbreak’ we identified 80 genomes from various studies that had only deposited reads with NCBI SRA, and which we reassembled for this study (see below).

For metagenomic search, we screened about 400 000 metagenomic read sets in a reduced representation known as *MinHash* signature [17] using wort (no version, unpublished, github.com/dib-lab/wort). These read sets represent a random sample from the entire NCBI SRA corpus, which we were limited to when this analysis took place (last accessed 1 June 2020). Hashing was performed using sourmash (v3.5) [18]. As the query we used the Kp-1 index genome (*k*=51, sampling rate 0.001) and manually reviewed all 15 hits reported with a threshold 0.01 Jaccard similarity, a measure that approximates average nucleotide identity (ANI) [17]. Most hits were synthetic consortia of microbes, assembled for an undetermined experimental purpose. Three hits were repeat stool samples from a single person, and we continued our analysis with the earliest sample (see Results).

### Strain-specific screening PCR

We then screened two culture collections (National Reference Centre for multidrug-resistant Gram-negative bacteria, Bochum, and Department of Medical Microbiology and Virology, Leipzig) for related isolates using a strain-specific marker PCR, designed using a proprietary, pangenome-based algorithm (nanozoo GmbH). Each 50 µl PCR reaction contained 10 µl template DNA, 2 µl 10 nM primer mix for each primer (primer 1: ATGCGTCCACGAAGAATTAT; primer 2: CATCGCCAAGATACTGTACA), 25 µl 2× polymerase master mix (Superfi II, Invitrogen) and 11 µl ultra-pure water. Thermal cycling consisted of initial denaturation at 98 C for 1 min followed by 35 cycles of denaturation at 98 C for 20 s, annealing at 55 C for 20 s, extension at 72 C for 1 min, followed by final extension at 72 C for 5 min.

### Data processing

Unless otherwise stated, default parameters were used. Of the 9409 collected genome assemblies, 1461 passed a minimum Jaccard similarity of 0.97 compared to the Kp-1 index genome (15.5 %, parameters: *k*-mer size 51 nt, scale 0.001). Jaccard similarity was computed using sourmash (see above). In a subsequent filtering step, 415 (4.4 %) were included for tree construction based on a minimum *in silico* DNA–DNA-hybridization threshold of 99.98 % computed using FastANI (v1.32) [19] as well as a minimum genome length of 5 Mb and an alignment of 90 % of the query genome to the Kp-1 index isolate (completed, circular), excluding all extra-chromosomal sequences. This alignment was performed using minimap2 (v2.17-r941) [20] with the asm5 option for an expected sequence divergence of 0.1 %. This sequential approach allows for laxer but computationally efficient methods with fewer constraints to screen many genomes in the beginning. Subsequently, the selection is refined using more computationally expensive methods. We conservatively removed 16 samples from the timetree because they did not fit the estimated molecular clock model, likely due to unidentified recombination (Table S3).

Isolates where only short reads could be obtained were assembled using shovill (v1.1.0, unpublished, github.com/tseemann/shovill). Metagenomic reads were preprocessed using fastp (v0.20.1) [21] and assembled using megahit (v1.2.9) [22]. All contigs with a minimum length of 2 kb were then mapped to the reference genome (Kp-1 index patient, VA13414, Table S1) using minimap2 (see above). The Nanopore sequencing data were basecalled using Albacore (v2.3.2, available from Oxford Nanopore Technologies) and adapters removed using Porechop (v0.2.3, unpublished, github.com/rrwick/Porechop). Genome hybrid assembly using long and short reads was performed using Unicycler (v0.4.6) [23].

Genome annotation was performed using prokka (v1.14.6) [24]. Annotation of *Klebsiella *-specific features was done using kleborate (v0.4.0-beta) [25]. Plasmids were annotated using abricate (v1.0.1, unpublished, github.com/tseemann/abricate) using the plasmidfinder database (version 2021-01-13) [26]. Antimicrobial resistance genes were annotated using the same programme with the *Comprehensive Antibiotic Resistance Database* (CARD, v3.1.2) [27]. Genes from toxin-antitoxin systems were searched using mmseqs2 (v12-113e3) [28] against the *Toxin-Antitoxin Database* (TADB) (v2) [29]. Phages were annotated using uv (v0.1, unpublished, github.com/phiweger/uv). Recombinant regions were annotated using gubbins (v2.4.1) [30]. Single nucleotide variant (SNV) calling was performed using the snippy workflow (v4.6.0, unpublished, github.com/tseemann/snippy), which proved the most accurate programme in a recent benchmark study [7]. Note that SNV calling was based on the assemblies of all samples in our collection; where only reads could be obtained from the literature, we assembled the data first (see above). In short, snippy simulates reads from input genomes and maps them to the provided reference using bwa (v0.7.17-r1188) [31], before calling variants with freebayes (v1.3.2, unpublished, github.com/freebayes/freebayes). Putative recombinant, repetitive and prophage regions were masked before SNV calling. Sites with SNVs were extacted using snp-sites (v2.5.1) [32].

### Reconstruction of time-dated phylogeny

A time-dated phylogeny was calculated using timetree (v0.7.6) [33], a maximum-likelihood-based approach starting from a core-genome SNV alignment. The derived mutation rate was scaled by the total genome size. Homoplasy was assessed using the treetime homoplasy function. The final tree and associated metadata were visualized using the microreact webservice [34]. Bootstrap support values were extracted from the guide tree, a prerequisite of the timetree, and calculated with raxml-ng (v0.9.0) [35].

### Analysis of genomic variants

A genome-wide association study was performed using pyseer (v1.3.7) [36] and included the aggregation of mutations across genes in a *burden test* [37]. Gene-set enrichment was performed using the Gene Ontology webservice (last accessed 1 April 2021) [38, 39]. Positive selection was assessed by first aligning all sequences for a particular gene using nextalign (no version, unpublished, github.com/nextstrain/nextclade). The multiple sequence alignment was then analysed using the BUSTED algorithm [40] as part of the HyPhy suite (v2.5.31) [41]. *In silico* folding of proteins was done using the trRosetta model (no version) [42].

### Plasmid containment and plasmid gene content

For plasmid containment, we first calculated one MinHash signature (target, T) for each of the four plasmids found in the Kp-1 index isolate, again using sourmash (*k*=21, scaled=100). Next, we sketched all isolate genomes (query, *Q*) with the same parameters. MinHash containment can then be calculated as the size of the union over the size of the target $\frac{|T|\cap |Q|}{|T|}$ . In other words, how much of each Kp-1 plasmid is contained in a query isolate genome, ranging from 0 (no containment) to 1 (all sampled *k*-mers are contained, i.e. target and query likely contain the same plasmid).

To explain the choice of *k-mer size* and sampling rate in MinHash-based genome similarity and containment calculations further: Generally, the larger the *k*-mer size, the more stringent the comparison, because as variation increases between two sequences, fewer *k*-mers are shared when fixing *k* to any particular value. For the genome filtering (see above), we thus chose a community-standard value for *k* to compare closely related sequences on the taxonomic scale of ‘strain’ (*k*=51). Species-level comparisons are usually conducted with *k* set to 31. Note that such values derive from experiments [17, 43], but there are no canonical values, and one has to experiment given the individual use case. For the plasmid containment analysis, we relaxed the *k*-mer size to 21 bases, which is commonly used to compare sequences where one expects more genetic variation, roughly at the level of ‘genera’ [43]. We justify this setting because compared to bacterial chromosomes, plasmids can present more genetic variation [44]. More genetic diversity requires a smaller *k*-mer size to approximate ANI accurately [17].

The *sampling rate* affects how many *k*-mers are sampled. The higher the rate, the more sensitive similarity and containment calculations are, but the less computationally efficient. Based on our experience and in line with values commonly used by the community, we typically use sampling rates of 1 in 10 000 for large metagenomes, 1 in 1000 for bacterial genomes, and 1 in 100 for small genomes and genomic elements like viruses and plasmids. We roughly target to sample at least 400 *k*-mers per sequence as this has been shown to suffice for average nucleotide identity (ANI) estimation [17].

Pangenome reconstruction for plasmids from hybrid assemblies (‘plasmidome’, *n*=28) was done using panaroo (v1.2.8) [45]. This resulted in a set of genes, each one representing an orthologous gene cluster. After translation into protein sequence, we searched for these genes in all outbreak genomes using mmseqs2 (v12-113e3) [28], resulting in a presence–absence matrix (Fig. 2b).

## Results

### *In silico* and PCR-based screening identifies hundreds of outbreak-related, contextual genomes

From 2010 to 2013, UHL experienced a large outbreak of a multiresistant, *bla*_KPC-2_-carrying Kp strain (hereafter referred to as ‘Kp-1’) of sequence type ST258, characterized by capsule type KL106 and O antigen (lipopolysaccharide, LPS) serotype O2v2. Overall, 105 patients were affected, and it took a multidisciplinary team many months to contain it [10] (Fig. S1). In 2018, we received 13 isolates from a *bla*_KPC-2_ Kp outbreak from the University Hospital Jena, located about 80 km from UHL (‘Kp-2’). We hypothesized that these were related to the Kp-1 outbreak at UHL due to the spatiotemporal proximity. A comparison of two genomes from Kp-1 and Kp-2, isolated from the respective index cases, showed that they were closely related. Kp-2 had the same sequence type (ST258), capsule type (KL106) and serotype (O2v2) as Kp-1. The genomes differed in only 69 SNVs, without larger rearrangements or differences in gene content. While within-hospital Kp outbreaks have been estimated to differ by fewer than 21 SNVs [5], we are unaware of recommendations for isolates further apart in space and time. Therefore, more ‘contextual’ genomes were needed to populate the genomic distance between Kp-1 and 2 and to fill the genomic ‘gap’. We, therefore, performed a comprehensive, multimodal screening, consisting of (1) a comprehensive literature search including manual extraction of genomes and metadata, (2) an *in vitro* screening of two German culture collections and (3) an *in silico* screening of publicly available genomic and metagenomic datasets.

In total, we obtained 9409 Kp genomes. Of those, 142 were collected during the Kp-1 outbreak from 105 patients [10], and 28 isolates were randomly selected for additional long-read sequencing (Nanopore) to obtain accurate plasmid reconstructions. The sampling aimed to uniformly represent all branches of the Kp-1 outbreak phylogeny. No long-read sequencing was performed on Kp-2 isolates, as we only obtained them later on in the analysis. A further ten isolates were identified in two culture collections through PCR-based screening using strain-specific primers (see Methods). The local UHL collection holds several hundred multiresistant Gram-negative bacteria, all recruited from the hospital over the last 20 years. The second collection is the German National Reference Center for multidrug-resistant Gram-negative bacteria in Bochum and holds an order of magnitude more isolates, which are subject to a standardized characterization. Sequencing of the ten isolates confirmed that all were closely related to Kp-1. The primers were designed using a proprietary algorithm (nanozoo GmbH) to recognize Kp-1 and close relatives but not other Kp strain genomes, e.g. different sequence types. Interestingly, the algorithm selected a putative intact prophage region as the most specific PCR template, and >99 % of genomes closely related to Kp-1 and selected for further analyses (see below) contained the target. Thus, even though phages are mobile, they can be remarkably stable across decades and still be used as effective markers [46].

The remaining 9257 genomes were collected from public sources. The majority was retrieved from NCBI *RefSeq* [16]. However, 80 isolate read datasets were only identified through a literature survey, as they did not have an associated genome assembly deposited. In addition, extensive metadata were extracted where available. Furthermore, we searched the index Kp-1 isolate in a *k*-mer database of over 400 000 metagenomic read datasets (SRA, NCBI, see Methods). We identified a single sample from an unpublished study of ICU patient colonization (NCBI, project ID PRJNA561398) where we could recover a closely related, metagenome-assembled Kp genome.

**Fig. 1.**
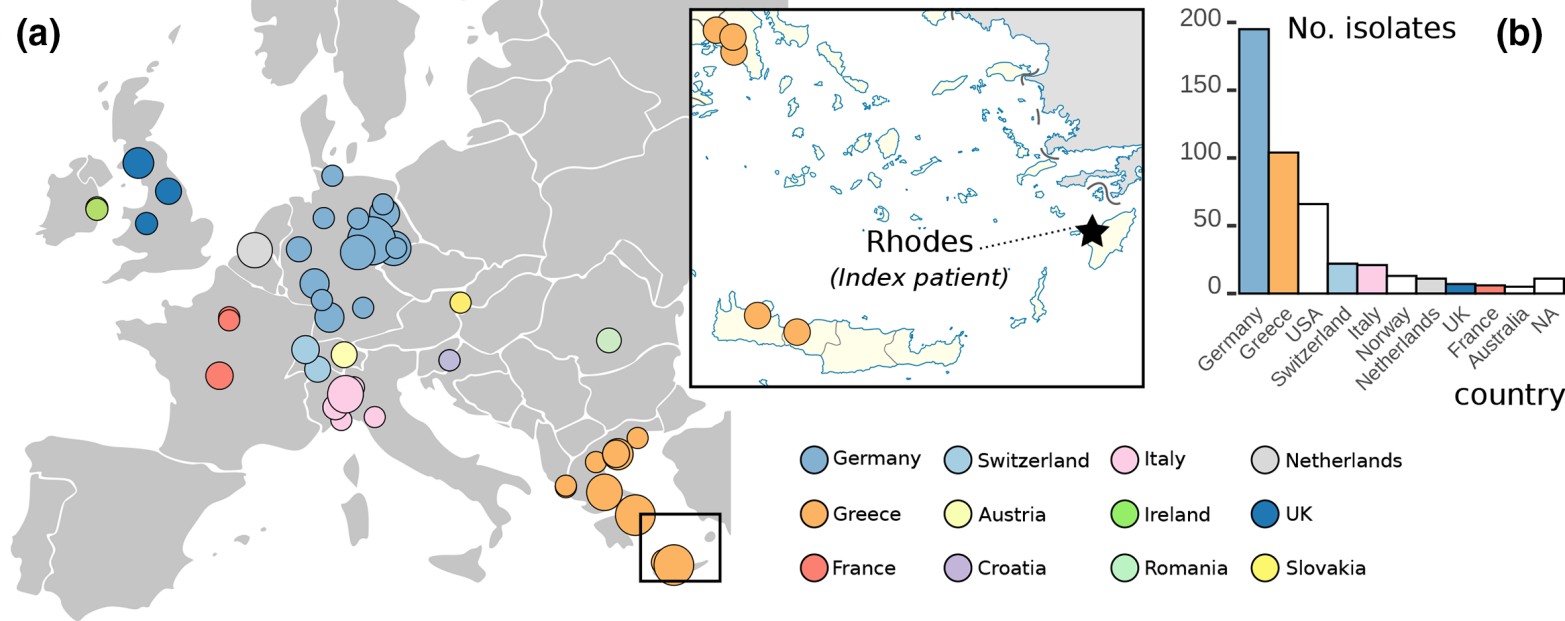
Geographic distribution of the genomes under study. (a) Location of isolation sources in Europe. Circle size is proportional to the number of genomes collected from this location. Leaves were coloured by country code. In the detailed map to the right, genomes found in Crete are shown (bottom). Our index patient was hospitalized in nearby Rhodes (star), and endemic transmission across these islands, which are connected by boat, is plausible. (b) Distribution of countries from which isolates were collected. Only countries with more than five isolates are depicted.

From the collected 9409 genomes, we wanted to select the ones most similar to the Kp-1 index genome. Altogether, 415 genomes (4.4 %) passed a tiered quality-control protocol (see Methods), resulting in a collection of high-quality genomes (ANI >99.98 %, alignment to Kp-1 index isolate >90 %) for further analyses. The 415 accepted genomes represent isolate and metagenomic read data, from a total of 28 studies and 16 countries (Fig. 1a, b, Table S1). Sequence type, capsule type and serotype of most selected isolates closely match Kp-1 (ST258, KL106, O2v2). Isolation dates span 15 years, from 2006 to 2018. Most isolates were recruited from European countries, particularly Germany and Greece. All but one isolate were clinical; that one was isolated from wastewater (NCBI, project ID PRJNA579879) [47]. Unfortunately, the metadata on isolation sources was sparse, and we did not collect this information for further analyses. Our impression, however, was that roughly half of the isolates were obtained through some kind of screening effort. Additional metadata, including data sources and associated publications, where available, can be found in Table S1.

### Time-dated phylogeny resolves outbreak origin and reveals hidden transmission

**Fig. 2.**
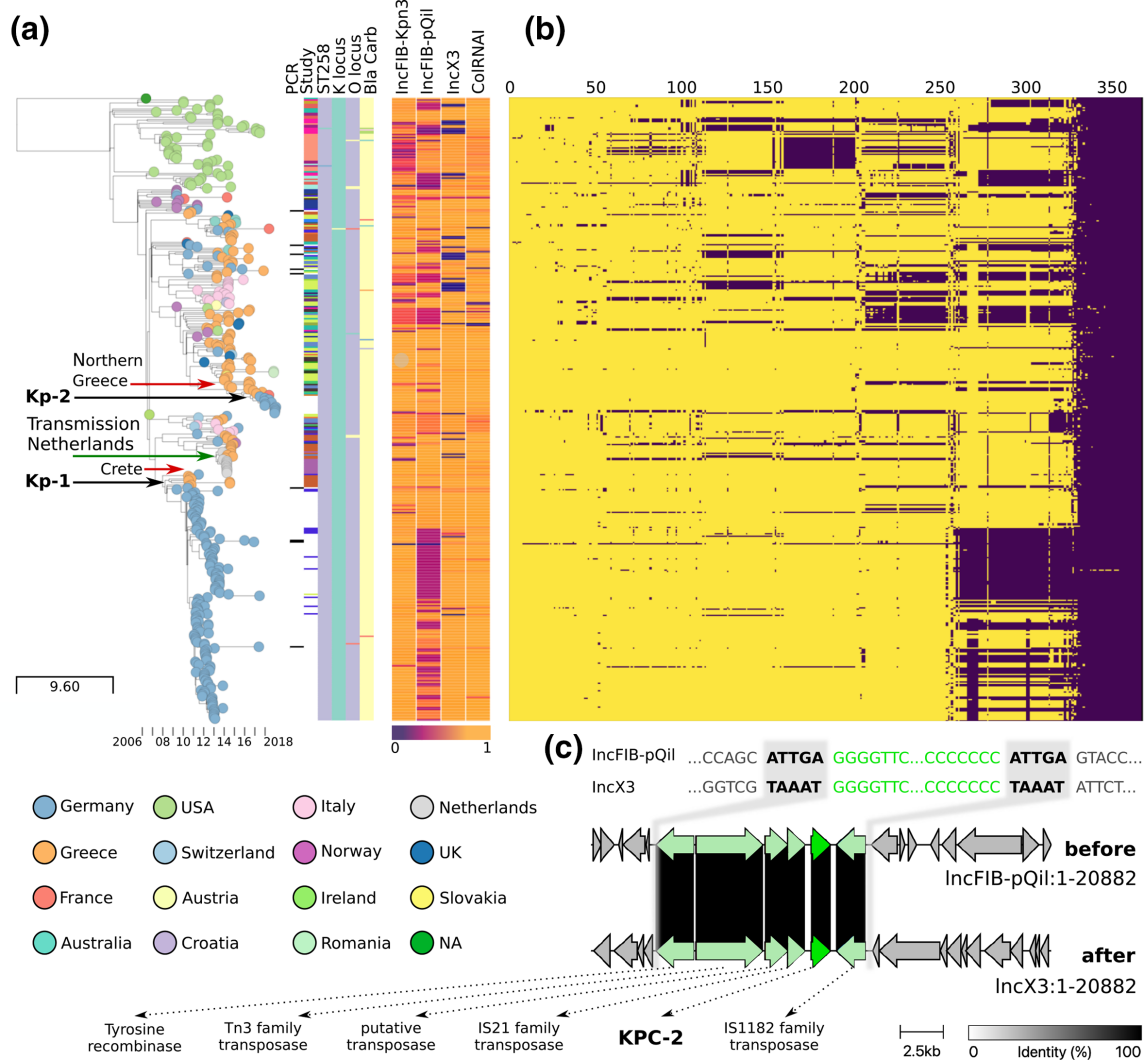
Time-dated phylogeny of 415 *bla*_KPC-2_ carrying Kp genomes and associated metadata blocks. For interactive exploration see visualization in microreact [34] (https://microreact.org/project/6bBfAYXswvY691LfbVytLT). (a) A timetree reveals how both outbreak strains, Kp-1 and Kp-2 (black arrows), most likely originated from southern and northern Greece (red arrows), respectively. Two scales are provided: SNV distance (left) and a timeline depicting year of isolation (right). In the leftmost metadata block columns, read from left to right, genomes are marked that have been identified using our strain-specific screening PCR. The second column indicates which study they were recruited from (white is our study, all of the other 28 colours are study-specific, see Table S1). The next four columns show the sequence type (purple is ST258), capsule type (turquoise is KL106), O antigen (LPS) type (purple is O2v2), and carbapenemase variant (‘Bla Carb’, yellow is *bla*_KPC-2_, other colours indicate either combinations with other carbapenemases like *OXA* or other *KPC* variants, see interactive plot). Note how the majority of genomes that pass our tiered filtering approach are homogenous in these features (see also Table S4). The following metadata block shows what fraction of each of the four plasmids in the Kp-1 index genome is contained in each isolate genome from our collection. Containment values range from 0 (blue, no containment) to 1 (orange, Kp-1 plasmid is wholly contained in the query isolate). (b) Matrix indicates presence (yellow) or absence (purple) of plasmid genes (columns) for each genome (row) in the phylogenetic tree. (c) Alignment of genes around the *bla*_KPC-2_ locus between the two plasmids IncFIB(pQil) (top) and IncX3 (bottom) shows a transposition event that allows shedding of IncFIB(pQil) while maintaining *bla*_KPC-2_ on the IncX3 plasmid, likely increasing host fitness. Transposed sequence in light green, *bla*_KPC-2_ in dark green. The 5 bp target site duplications (bold sequence) indicate that Tn*4401* (green sequence) moved through transposition rather than recombination [58].

We observed 69 SNVs between the genomes of Kp-1 and Kp-2. However, relating isolates based on mutation rates alone can be misleading because rates of Kp are variable and thus difficult to estimate [48–50]. Therefore, to see whether Kp-2 descended from Kp-1, we constructed a time-dated phylogeny based on an alignment of 3720 core SNV sites (total alignment length 5 384 856 sites) from the 415 genomes in our filtered collection (Fig. 2). This phylogeny clearly shows that Kp-2 is not a direct descendant of Kp-1. The tree topology did not change when we fixed it to mutation rates reported in the literature instead of estimating the mutation rate from the data.

The index patient’s travel history and symptom onset led to the hypothesis that the origin of the Kp-1 outbreak was a Kp strain imported from Rhodes, an island in southern Greece and a popular tourist location for German travellers. After being acutely hospitalized there, the patient was transferred to UHL, where *bla*_KPC-2_ was detected for the first time in the patient’s medical history. A Greek origin seemed plausible because of the travel and the high prevalence of carbapenemase-carrying strains in Greece [51]. However, this hypothesis could not be substantiated with this data alone, and no supporting isolate genomes from Greece were available [10]. We identified several closely related genomes from Crete [52], a neighbouring island of Rhodes (see detailed map in Fig. 1a), which populate the timetree around the time of the start of the Kp-1 outbreak (Fig. 2a, black arrow ‘Kp-1’). With frequent travel by boat between these islands, it is plausible that an ancestor of Kp-1 was circulating in this region. Interestingly, the originating strain for the Kp-2 outbreak also seems to have come from Greece, albeit from northern provinces. Here, we could identify closely related genomes from two studies [5, 53] (Fig. 2a, black arrow ‘Kp-2’). We even identified a third transmission from Greece to mainland Europe, with a strain from northern Greece causing an outbreak in the Netherlands [54] (Fig. 2a, green arrow). The authors of the corresponding study did not identify this origin because they limited their investigation to local cases, supporting our argument for an integrative approach across study borders. All nodes in the tree where these transmissions out of Greece appeared had over 95 % bootstrap support. However, it is important to consider potential sampling bias when inferring origins. While we identified many samples from Greece (Fig. 1b), the screening methods were blind towards genome origin and considered an exhaustive set of Kp genomes. Furthermore, several studies have described the high prevalence of carbapenemase-carrying Kp in southern Europe [51]. Therefore, we argue that the large number of Greek samples likely represents the true distribution of *bla*_KPC-2_ Kp and is not an artefact of sampling bias, though it cannot be ruled out completely.

Shortly after the outbreak onset, a systematic screening of patients on hospital admission was established. From then on, we did not detect any new Kp-1 isolates on admission but only after, which argues against a re-importation from Greece. In many cases, a causal transmission chain could be reconstructed [10]. As the Kp-1 outbreak unfolded, local health authorities assumed that the outbreak was likely not limited to one hospital. They based their assessment on the long duration and the large number of patients involved in the outbreak, with frequent transfers to and from the hospital as a tertiary care centre. While these factors make non-local transmission more likely, no evidence was available to support this hypothesis. Surprisingly, we identified 13 isolates that were collected outside of UHL, but are part of the Kp-1 outbreak (Fig. 2a, Table S2). Most of them come from the same federal state that UHL is in, but several were isolated in other states hundreds of kilometres away. We did not detect descendant isolates of the Kp-1 outbreak in other countries. The Kp-2 outbreak seems to have been contained within the affected hospital, as no published genomes were found in other places. On an international level, the data supports repeated introduction of KPC-carrying Kp strains from Greece, likely due to it being a popular travel site. In fact, travel-related carbapenemase-producing Enterobacterales have been recognized as an important source of resistance transmission [55]. The above described hidden transmission events would not have been observed without integration of data across study borders, and illustrate the value of our approach.

### Carbapenemase preservation in spite of frequent plasmid changes

Plasmids serve many functions, but a central one is as a gene-delivery platform [44]. Their payload is manifold, and here includes the *bla*_KPC-2_ carbapenemase. However, to the host genome, plasmids can come at a considerable fitness cost, which creates pressure to remove them unless they provide a selection advantage [44]. At the same time, plasmids resist removal through, e.g. toxin-antitoxin systems and compete with rivalling plasmids [44]. In search of persistence in the host, frequent changes to the genetic material of plasmids can be observed [56]. In the Kp-1 index genome, we found four types of circular plasmid using Nanopore-based hybrid assembly: IncFIB(Kpn3), IncX3, ColRNAl and IncFIB(pQil), the latter carrying *bla*_KPC-2_.

Plasmid reconstruction is often difficult for short-read-based assemblies due to assembly fragmentation. Thus, comparing plasmids between isolates usually means comparing sets of putative plasmid contigs. However, if any set is incomplete, such an analysis can be misleading. We, therefore, decided to use the newly sequenced, hybrid-assembled, circular plasmid reconstructions from the Kp-1 index genome as a reference and determined what fraction of these plasmids was contained in any of the 142 Kp-1 isolates that Kaiser *et al*. sequenced in 2017 (Fig. 2a) [10]. Containment here refers to MinHash containment (see Methods). To complement this data, we aggregated the plasmid-encoded gene content (‘plasmid pangenome’) across 28 Nanopore-sequenced isolates, leveraging the superior ability of this technology to reconstruct plasmids. We then determined whether these genes were present in all members of our isolate collection (Fig. 2b). The contextual genomes show repeat loss of IncFIB(pQil) (109 Kb) in phylogenetically distinct isolates, which likely constitute independent events (Fig. 2b). Interestingly, besides *bla*_KPC-2_ and the extended-spectrum beta-lactamase (ESBL) TEM, we also found a toxin-antitoxin system on IncFIB(pQil) (*vagC* and *vagD* [57]), which can prevent the host from shedding the plasmid. Nevertheless, we observed the complete loss of IncFIB(pQil) during the Kp-1 outbreak. About one-third of the Kp-1 outbreak genomes were affected, initially confusingly so because this plasmid carried the *bla*_KPC-2_ carbapenemase. However, we found one isolate with two *bla*_KPC-2_ copies, one on IncFIB(pQil) and IncX3 (43 Kb), respectively. The only other resistance gene on IncX3 was the SHV ESBL. As *bla*_KPC-2_ is carried on the Tn*4401* transposon, it could be copied from IncFIB(pQil) to IncX3 (Fig. 2c). Tn*4401* moving between plasmids was the result of transposition and not recombination, which can be inferred from 5 bp target site duplications (Fig. 2c) [58]. After this transposition event, the host could discard the IncFIB(pQil) plasmid, which likely confers a fitness advantage, but retain *bla*_KPC-2_ simultaneously.

### Contextual genomes reveal positive selection of virulence and resistance genes

Modified lipopolysaccharides often cause colistin and, more generally, polymyxin resistance (PR). They result in a positive charge to the bacterial membrane that repels polymyxins [12]. Several proteins are involved, though *‘the exact mode of action of polymyxins still remains unclear’* [12]. For 171 of the 415 genomes in our collection (41.2 %), we were able to assess from the original records and publications whether the isolate was colistin-resistant or not (Table S2). Where minimum inhibitory concentration (MIC) measurements were available, breakpoints by the EUCAST committee (v11) were used to classify isolates into colistin sensitive and resistant.

We found several known genes involved in PR to be mutated. Overall, 60 of the 171 isolates (35.1 %) with phenotype data were colistin-resistant. In 39 out of these 60 cases (65 %) we found a truncated or missing *mgrB* gene product, a negative regulator of the *PhoPQ* signalling system [59]. Interestingly, we detected an incomplete *mgrB* gene product in eight isolates reported as colistin-sensitive. *mgrB*-mediated resistance can occur rapidly: in the Kp-1 outbreak, we identified three different *mgrB* loss-of-function mutations (Fig. S2). Furthermore, we found frequent truncations in *pmrB* and non-synonymous but not truncating or missense mutations in *phoQ* (17 UM) and *phoP* (9 UM), all regulatory proteins involved in LPS modification [60]. These ‘canonical genes’ [59] cause PR by acting on the outer membrane. We did not detect the plasmid-encoded *mcr-1* gene, which encodes a transferase that modifies lipid A and thereby causes PR [12].

**Fig. 3.**
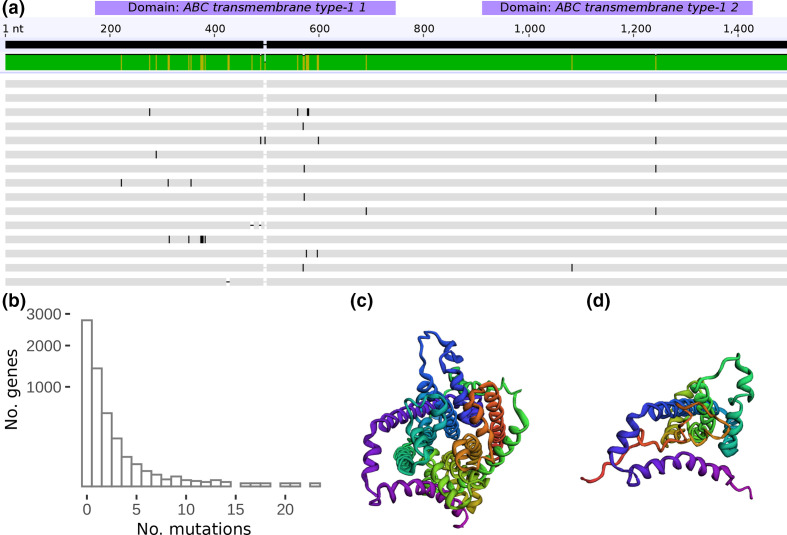
Positive selection of the inner membrane ABC transporter permease *ynjC*. (a) Multiple sequence alignment of representative haplotypes of the nucleotide sequence of *ynjC*. Most mutations occur between positions 75 to 730, which includes both transmembrane and topological domains. Three of those haplotypes lead to premature stop codons. (b) Distribution of unique mutations observed in all genes. As expected by the estimated mutation rate of 0.68 mutations per Mb per year, most genes remain unchanged over the ten years that our study covers. Several genes, however, accrue over 20 unique mutations across 415 genomes. (c) Three-dimensional (3D) protein structure of the *ynjC* permease. In the centre is the pore through which small molecules are shuttled. (d) 3D protein structure of a truncated form of the protein [same orientation as (c)], created through a premature stop codon. Clearly, the channel structure is lost, and the protein is likely dysfunctional.

To identify other genomic regions associated with PR, we first performed a genome-wide association study (GWAS) based on SNVs, small insertions and deletions. This analysis did not return a significant result after correcting for population structure (*P>*0.05), neither when considering each SNV individually nor when aggregating SNVs over genes in a so-called *burden test* [37]. This failure might be due to technical limitations of GWAS, especially in light of few genomes [61] or strong population structure [62, 63]. Furthermore, while single SNVs can induce colistin resistance [64], PR is generally assumed to be a polygenic phenomenon [12]. To test which genes were mutated more than expected, we first aggregated unique haplotypes for each gene across all genomes, similar to a *burden test* [37]. To conservatively correct for population structure, we counted mutations only once per position in the reference genome. Recombinant sites, putative phages and sites within repetitive sequences were excluded. This procedure would not detect convergent evolution where mutations arise in the same position in two different clades. However, we did not detect any homoplastic mutations outside of recombinant regions. At a mutation rate of 0.68 per Mb per year and 5.5 million bases' genome size, we expect about four mutations per year in the Kp genomes under study. Consequently, most of the roughly 4000 genes in the genome remain unaltered, even in the ten years our genome collection is distributed across (Fig. 3b). Conversely, if we observe more mutations than expected in a gene, this might indicate evolutionary selection.

We, therefore, ranked genes by the number of unique mutations (UM) per gene. We define unique mutations as specific to a single gene and position. We used the number of UMs per gene as a heuristic to rank and prioritize genes for further analyses. Note that if an isolate presents a mutation at a particular position in some gene like *gyrA*, all descendants will likely share this mutation. However, we only count this mutation as a single UM for *gyrA*; only mutations in other positions in the gene would increase the UM count after that. A gene-set enrichment of all genes with 10 UMs (*n*=15) showed two overrepresented biological processes. For one, the phosphorelay signal transduction system was enriched (19.8-fold, *P*=0.001) [65], which is known to be implicated in PR [12]. Furthermore, genes associated with nitrate assimilation were enriched (41.8-fold, *P*=0.001), which to our knowledge has neither been described nor could we assess the biological significance of this finding.

We tested the top-ranking genes with the highest number of unique mutations for gene-wide evidence of episodic positive selection. For each candidate gene, we used a random-effects framework to pool evidence across multiple sites and thereby increase statistical power [40]. All genes discussed hereafter exhibited significant positive selection (*dN/dS>*1, likelihood-ratio test, *P*=0.05). We found two positively selected genes that affect virulence: the transcriptional activator *cadC* (18 UM) has been linked to increased Kp colonization [66] and *fimH* (16 UM) is a critical virulence factor in urinary tract infection, a common complication of Kp colonization [67]. Note that *fimH* has a direct role in epithelial binding, and allele switching via homologous recombination is common [68].

Recently, colistin has also been found to target the inner cytoplasmic membrane [69]. Interestingly, we identified a highly mutated inner-membrane ABC transporter permease [70] under strong positive selection (all detected mutations non-synonymous), named *ynjC* (21 UM, Uniprot, P76224). Proteins of this group utilize ATP to import many small molecules such as nutrients and antibiotics [71–73]. Mutations in permeases have been shown to ‘lock’ the transporter in one of its two states [74, 75], such as inward-facing [76], disrupting the shuttle function [77]. Additionally, we found three mutations that caused premature stop codons and subsequent dysfunctional proteins (Fig. 3c, d). Most mutations accumulate in a region between residues 75 to 730, spanning both transmembrane and topological domains (Fig. 3a). In 12 isolates with *ynjC* mutations, 7 (58.3 %) were resistant to colistin; however, for none of the haplotypes with premature stop codons, phenotype data could be obtained, and future functional validation is needed. Nevertheless, ABC family transporters have been proposed to transport nascent core-lipid A molecules across the inner membrane [78], with a putative effect on colistin resistance. They have also been proposed as an antibiotic target [79]. We thus argue that the *ynjC* permease could have a role in PR.

## Discussion

Genomic surveillance is a powerful public health tool to reduce the spread of resistant bacteria. We show that genomic meta-analysis of outbreak genomes can provide important contextual information when interpreting local outbreaks. To construct the context, we employed both *in vitro* and *in silico* search methods to aggregate more than 400 genomes to supplement the local outbreak under investigation, screening more than 10 000 genomes and nearly half a million metagenomes in the process. As a result, we discovered critical epidemiologic details that would have been missed in a traditional outbreak study focusing on local data only. For example, we determined the likely source of the Kp-1 outbreak, its relation to an outbreak at a nearby institution, and it being an instance of the repeated introduction of *bla*_KPC-2_ Kp isolates into mainland Europe from Greece. We also identified isolates from other studies that are direct descendants of Kp-1.

We then illustrated the plasmid dynamics across our genome collection. We found frequent loss of genetic material associated with IncFIB(pQil)-type plasmids, even though they often carry the *bla*_KPC-2_ gene. We resolved this paradox by showing how *bla*_KPC-2_ can still be preserved in the host: the carrier transposon is first transferred to another plasmid before IncFIB(pQil) removal from the host. Such spread of *KPC* genes between plasmids over short time scales has been documented previously [80, 81].

Besides phylogenomic insights, our context-enriched genome collection informs about adaptation to selective pressure. For one, we found several positively selected genes that are known to mediate, e.g. colistin resistance. We also discovered positive selection of the inner-membrane transporter *ynjC* together with an overrepresentation of mutated gene copies in colistin-resistant isolates. However, future experiments will have to validate if an effect on colistin resistance can indeed be shown, e.g. by introducing loss-of-function mutations using CRISPR [59].

The pathogens in our filtered dataset can be assumed to be under similar selective pressures, as all but one were isolated from hospital patients. All isolates carry a carbapenemase, where few antibiotics such as colistin remain as a rational treatment option, sometimes combined with rifampicin for synergy [82]. Based on standard clinical practice, we argue that many patients will have received colistin to either decolonize them or treat an infection. Kp colonization has been described as a risk factor for subsequent infection [83], and colonization can persist for years [84], increasing the chance of infection. Indeed, it is likely that Kp isolates will evolve to facilitate long-term carriage. In the Kp-1 outbreak, about half the patients with *bla*_KPC-2_ detection presented with clinical disease, mainly pneumonia, and sepsis, and were subsequently treated with colistin either alone or in combination [8]. We assume that these numbers apply to other hospitals as well. In the absence of infection, decolonization is sometimes attempted using colistin. While we observed low efficacy and rapid resistance induction [85], colistin continues to be used for decolonization [86]. In the metadata collected in this study, we did not find any treatment descriptions other than anecdotal ones.

Many genomes in our collection were assembled using different and often insufficiently documented methods. It is thus possible that observed mutations are technical artefacts. However, most genomes were reconstructed from accurate short reads. Therefore, we think the effect of spurious SNVs (in contrast to structural variants, which are a problem with this method) is negligible. However, it must be taken into account as a potential source of error. We also chose a conservative approach to control for unobserved effects other than technical error, such as geography, or sampling site, limiting our analyses to unique mutations and only counting mutations once per gene and locus across the entire dataset.

Colistin resistance can be unstable and transient [87]. Also, colistin resistance can be observed in the absence of explanatory genetic changes [88]. A genome-wide association study (GWAS) of this trait is thus challenging, and in fact, our analysis did not yield any significant target that could predict colistin resistance. The heterogeneous genome collection also complicates the GWAS. Because we often start from pre-assembled genomes, we have no control over the sequencing and assembly process. Similarly, we have no way to check the accuracy of the sparse metadata. Also, we completely lack data on which drugs or other selective pressures most isolates have undergone and can only infer them. Thus, our GWAS using contextual genomes was limited in its explanatory power.

Several components are still missing until we can analyse putative outbreak genomes in a real-time, integrated surveillance system. The main bottleneck, counter-intuitively, is not sequencing but data management and bioinformatics [89]. For example, there is no common repository for bacterial outbreak metadata in active use by the community. We manually aggregated metadata from 28 studies, which frequently involved squinting at low-resolution images to extract, e.g. data on colistin resistance. For most genomes, important information besides the year and country of isolation was missing. Without this metadata, the sequenced genomes cannot easily be integrated into any analysis other than the one they were originally sequenced for. This could be aided in the short term if authors published Supplementary Material giving genome accessions alongside all relevant isolate data, genotypes and phenotypes explored in the study.

Also, more sophisticated tools for outbreak genome sharing are needed [90]. Most outbreak studies appear 1 to 2 years after the outbreak took place (personal observation). However, by then, the value of the results is primarily academic. Only prospective data analysis [91] in real-time would enable a practical outbreak response. A recent example of this is nextstrain, where the virus genomics community converged on a set of protocols and databases [92], which allowed a data-driven public health response. When combined with real-time sequencing of bacterial genomes [93], this set of technologies could substantially improve outbreak response.

Our metagenomic screening returned an isolate closely related to Kp-1, sampled on a different continent. Generally, we think that metagenomic screening for isolates of interest holds great promise. The construction of the required index for an entire read collection such as the NCBI SRA is challenging, as is the design of an easy search interface. However, once operational, such a system allows resistance and virulence tracking in, e.g. mobile genetic elements across species and habitats, which is especially relevant for pathogen outbreak investigations.

## Supplementary Data

Supplementary material 1

Supplementary material 2

Supplementary material 3

Supplementary material 4

Supplementary material 5

Supplementary material 6
