# Supplementary material for: Context-aware genomic surveillance reveals hidden transmission of a carbapenemase-producing Klebsiella pneumoniae
Source: Microb Genom. 2021 Dec 16;7(12):000741. doi: 10.1099/mgen.0.000741 (PMC8767333; doi:10.1099/mgen.0.000741)
Supplement: Supplementary material 1 [file mgen-7-0741-s001.pdf]

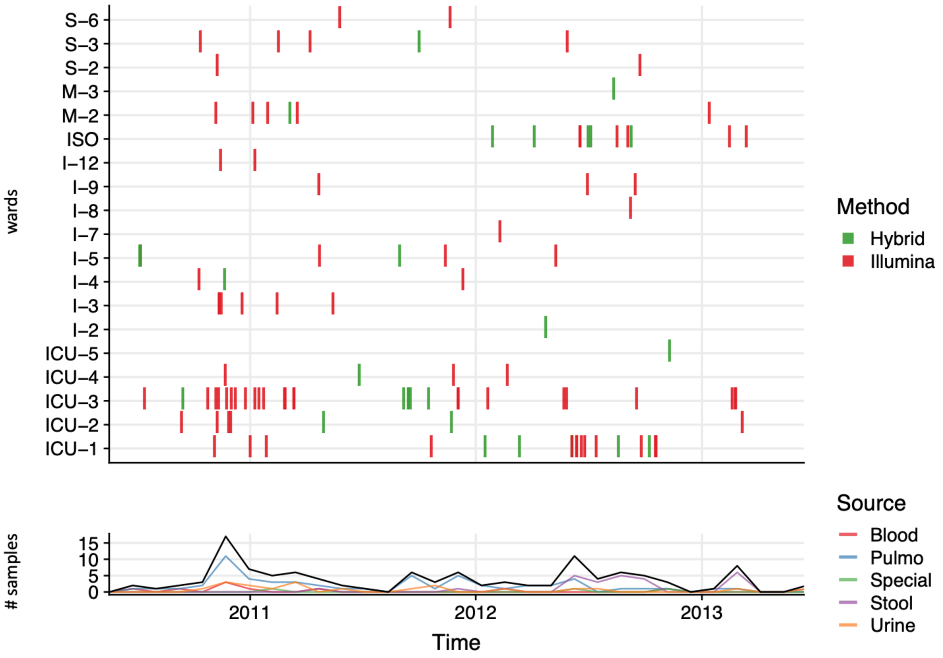

Figure S1: *bla*$_{\text{KPC-2}}$ Kp-1 case distribution. All isolates were sequenced using short reads. For long-read sequencing, 28 representative samples were selected, uniformly distributed across time and space.

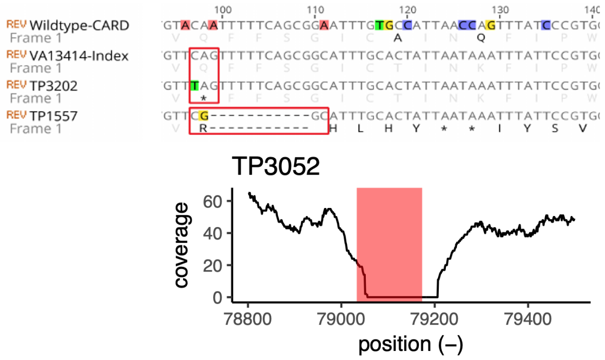

Figure S2: **(Top)** Detail from a multiple sequence alignment of representative *mgrB* sequences (partial sequence displayed). Three different variants caused *mgrB* truncation in the Kp-1 outbreak, two of which are illustrated here. From top to bottom: Reference *mgrB* sequence from the CARD database, gene sequence from the Kp-1 index isolate, *mgrB* where a SNV causes a premature stop codon, gene sequence with an 11 bp deletion and subsequent frame-shift. **(Bottom)** One isolate presented a complete loss of *mgrB*, as could be validated by mapping the short reads from this isolate to the *mgrB* locus in the Kp-1 index genome.
